# Protein Induced by Vitamin K Absence or Antagonist‐II: Significantly Elevated in Obstructive Jaundice and Sepsis Patients Without Hepatocellular Carcinoma

**DOI:** 10.1002/jcla.70128

**Published:** 2025-11-13

**Authors:** Ke Zhang, Xiarui Ye, Xianran Fu

**Affiliations:** ^1^ Department of Clinical Laboratory, Union Hospital, Tongji Medical College Huazhong University of Science and Technology Wuhan Hubei China

**Keywords:** alpha‐fetoprotein, hepatocellular carcinoma, protein induced by vitamin K absence or antagonist‐II, sensitivity, specificity

## Abstract

**Background:**

PIVKA‐II is a promising diagnostic and prognostic biomarker for HCC. However, a particular PIVKA‐II variant can be abnormally elevated in patients with obstructive jaundice or sepsis, complicating its interpretation in HCC conditions.

**Methods:**

This study aimed to investigate the distribution and positive rates of serum PIVKA‐II and AFP in patients with obstructive jaundice or sepsis, and to explore their relationships with laboratory tests, particularly coagulation function indexes. The receiver operating characteristic (ROC) curve was used to determine the cut‐off, specificity and sensitivity of PIVKA‐II in diagnosing HCC.

**Results:**

In patients with obstructive jaundice and sepsis, PIVKA‐II was significantly positively correlated with PT and INR. When the cut‐off was 42.17 mAU/mL, the sensitivity and specificity of PIVKA‐II in diagnosing HCC were 74.69% and 70.17%, respectively. There was no difference in PIVKA‐II concentration between HCC and obstructive jaundice. PIVKA‐II levels in obstructive jaundice and sepsis were significantly higher than those in healthy controls. Except for healthy individuals, the positive rate of serum PIVKA‐II was significantly higher than that of AFP among the other three groups, and patients with obstructive jaundice had the highest positive rate of PIVKA‐II.

**Conclusions:**

The PIVKA‐II produced by HCC may differ from that produced by obstructive jaundice and sepsis. Given that PIVKA‐II levels are abnormally elevated in patients with obstructive jaundice and sepsis, the results should be interpreted with caution in patients with HCC combined with these diseases.

## Graphical abstract

The PIVKA‐II produced by HCC may differ from that produced by obstructive jaundice and sepsis. Given that serum PIVKA‐II levels are abnormally elevated in patients with obstructive jaundice and sepsis, the results should be interpreted with caution in patients with hepatocellular carcinoma combined with these diseases.

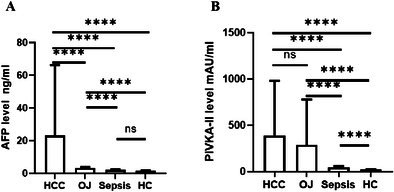

**Abbreviations**
AFP: alpha‐fetoprotein
APTT: activated partial thromboplastin time
CRP: C‐reactive protein
DCP: des‐c‐carboxy prothrombin
HCC: hepatocellular carcinoma
INR: international normalized ratio
PCT: procalcitonin
PIVKA‐II: protein induced by vitamin K absence or antagonist‐II
PT: prothrombin time

## 1 Introduction

Liver cancer represents a significant global health challenge, with projections indicating that it will affect more than 1 million individuals annually by 2025. Hepatocellular carcinoma (HCC), the most common type of primary liver cancer, accounts for approximately 90% of cases and is the fourth leading cause of cancer‐related death worldwide. Given its high mortality rate, early detection through screening programs is essential, as timely diagnosis enables effective treatment and improves long‐term disease‐free survival [1, 2].

Current guidelines recommend performing abdominal ultrasound every 6 months for the surveillance of patients with cirrhosis. However, it has been established that ultrasound is insufficiently reliable for detecting hepatocellular carcinoma at an early stage, with a sensitivity of only 63% in this setting [3, 4]. Thus, additional biomarkers for early HCC detection are needed to complement ultrasound. Alpha‐fetoprotein (AFP) is the most widely used serum marker for HCC surveillance globally. Previous studies have reported that serum AFP demonstrates a sensitivity of 39%–65% and a specificity of 76%–94% for detecting HCC, which remains suboptimal for clinical application. Protein induced by vitamin K absence or antagonist‐II (PIVKA‐II), also called des‐c‐carboxy prothrombin (DCP) initially identified in 1984 as a specific biomarker for HCC [5], has emerged as a promising diagnostic and prognostic tool for HCC surveillance, including in early‐stage and AFP‐negative disease [6, 7]. Moreover, elevated PIVKA‐II serum levels and increased tissue expression have been associated with microvascular invasion [8], a major risk factor for tumor recurrence and mortality in HCC, though a consensus definition remains lacking [9].

Prior evidence has established the role of PIVKA‐II in screening and diagnosing HCC. However, it should be noted that PIVKA‐II can be elevated even in the absence of HCC, particularly among populations targeted for surveillance and pending diagnosis. PIVKA‐II levels are elevated in the presence of vitamin K deficiency [5, 10]. Additionally, antibiotics containing an *N*‐methylthiotetrazole substrate, obstructive jaundice (associated with pancreatic head cancer, biliary tract cancer, or benign stricture of the bile duct), alcohol abuse, and warfarin ingestion can lead to false positive PIVKA‐II results [10, 11, 12]. Therefore, we conducted a study involving patients with obstructive jaundice or sepsis to investigate the distribution and positive rates of PIVKA‐II and AFP and their relationships with laboratory tests.

## 2 Materials and Methods

### 2.1 Patient

This cross‐sectional study included patients with obstructive jaundice and sepsis, who were recruited from the departments of infectious diseases, respiratory and critical care medicine, interventional radiology, and hepatobiliary surgery at Wuhan Union Hospital. The study was approved by the Ethics Committee of Wuhan Union Hospital (the approved number 0887).

**FIGURE 1:**
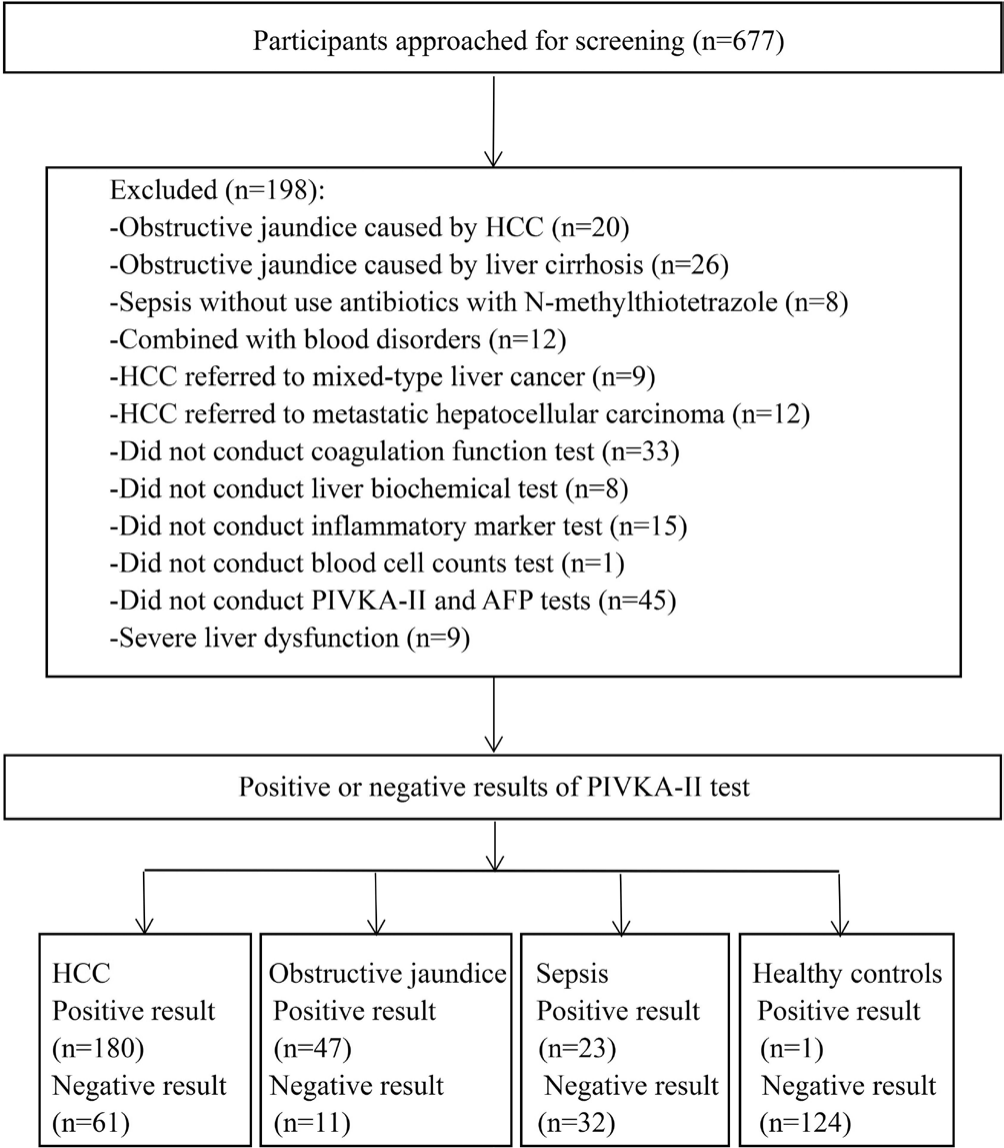
Flowchart of participants through study.

The obstructive jaundice cohort (*n* = 58) included patients with the following underlying conditions: pancreatic head cancer (*n* = 8), cholangiolithiasis (*n* = 4), cholangiocarcinoma (*n* = 40), cholestatic hepatitis (*n* = 4), and gallbladder adenocarcinoma (*n* = 2). Among these, 24 patients received multivitamin supplementation during hospitalization. Patients with sepsis (*n* = 55) who received antibiotics containing *N*‐methylthiotetrazolium substrates included pneumonia (*n* = 18), liver abscess (*n* = 40), and abdominal infection (*n* = 2), of which five cases had pneumonia and liver abscess co‐infection. Of these, 25 patients received multivitamin supplementation during hospitalization. Patients with liver cirrhosis, blood diseases, primary hepatocellular carcinoma, mixed primary liver cancer, metastatic hepatocellular carcinoma and severe liver dysfunction were excluded. A total of 241 patients with primary hepatocellular carcinoma and 125 healthy individuals were selected as the control group (Figure 1).

### 2.2 Measurements

Individuals included underwent blood sampling for liver biochemical tests, including ALT, AST, ALP, GGT, albumin, bile acids, total bilirubin, and conjugated bilirubin, as well as inflammatory markers (C‐reactive protein, procalcitonin), coagulation function parameters (PT, INR, APTT), blood cell counts (WBC, neutrophils), and tumor markers (PIVKA‐II, AFP). All measurements were conducted using standardized laboratory techniques at Wuhan Union Hospital.

Serum AFP and PIVKA‐II concentrations were measured by chemiluminescence microparticle immunoassay using the Abbott Architect i system. In this study, the established normal cut‐off values for these makers were as follows: AFP < 7.78 ng/mL and PIVKA‐II < 40.0 mAU/mL.

### 2.3 Statistical Analysis

Laboratory test concentrations were characterized using the median and interquartile range (IQR). Variables without a normal distribution were analyzed using the Mann–Whitney *U* test. Categorical parameters were evaluated using either the Chi‐Square test or Fisher's Exact Test. The statistical analyses were performed using SPSS software (version 22). Pearson correlations were determined among PIVKA‐II, AFP, and other variables. For the purpose of analysis, all laboratory values below or above the limit of detection for a particular assay were recorded as the lowest or highest value for that assay. All *p* values were two‐sided.

## 3 Results

One hundred and twenty‐five healthy individuals and 354 patients were enrolled in this study, including 58 with obstructive jaundice, 55 with sepsis, and 241 with primary hepatocellular carcinoma. Laboratory values for the study cohort are summarized in Table 1.

**TABLE 1:** Clinical and laboratory characteristics of individuals.

	Obstructive jaundice patients	Sepsis patients	Hepatocellular carcinoma patients	Healthy individuals
*N*	58	55	241	125
Age, years	60 (55–68)	61 (50–67)	57 (51–67)	58 (52–68)
PT, s	13.6 (12.8–15.5)	14.2 (13.7–15.2)	13.9 (13.4–15.0)	12.4 (11.8–13.5)
INR	1.07 (0.97–1.28)	1.10 (1.06–1.21)	1.09 (1.03–1.21)	0.95 (0.87–1.18)
APTT, s	36.4 (34.3–41.1)	39.1 (36.0–42.6)	38.2 (35.7–41.2)	32.4 (29.6–38.4)
AFP, ng/mL	3.3 (2.2–4.7)	2.0 (1.1–2.9)	23.1 (3.6–1713.8)	1.5 (1.2–3.0)
PIVKA‐II, mAU/mL	288.0 (45.9–1885.1)	33.7 (21.0–63.5)	388.2 (38.8–4109.2)	21.7 (15.5–25.1)
ALT, U/L	90 (49–149)	41 (27–77)	30 (22–45)	24 (9–36)
AST, U/L	96 (57–138)	32 (20–55)	40 (30–59)	22 (8–35)
GGT, U/L	320 (153–558)	None	78 (35–157)	18 (5–30)
ALP, U/L	324 (174–475)	None	112 (80–148)	72 (30–118)
Albumin, g/L	31.8 (28.4–35.3)	None	35.1 (31.6–38.6)	45.1 (38.6–49.6)
Total bilirubin, μmol/L	208.1 (72.3–318.4)	None	15.3 (11.4–22.2)	14.1 (6.3–18.4)
Conjugated bilirubin, μmol/L	121.0 (47.4–199.2)	None	6.7 (5.0–10.1)	4.7 (4.1–7.0)
Bile acids, μmol/L	69.4 (15.5–159.5)	None	10.8 (5.4–25.9)	5.8 (2.4–8.9)
CRP, mg/L	None	75.1 (30.3–131.5)	None	None
PCT, ng/mL	None	0.93 (0.12–5.78)	None	None
WBC, 10^9^/L	None	10.15 (6.02–13.04)	4.55 (3.40–6.09)	5.55 (3.65–7.12)
Neutrophil, 10^9^/L	None	8.19 (4.05–10.50)	2.63 (1.80–3.79)	4.27 (2.20–5.32)

*Note:* Data are presented as median and interquartile range (IQR).

Abbreviations: AFP, alpha‐fetoprotein; ALP, alkaline phosphatase; ALT, alanine aminotransferase; APTT, activated partial thromboplastin time; AST, aspartate aminotransferase; CRP, C‐reactive protein; GGT, gamma‐glutamyl transferase; INR, international normalized ratio; PCT, procalcitonin; PIVKA‐II, protein induced by vitamin K absence or antagonist‐II; PT, prothrombin time; WBC, white blood cell.

**FIGURE 2:**
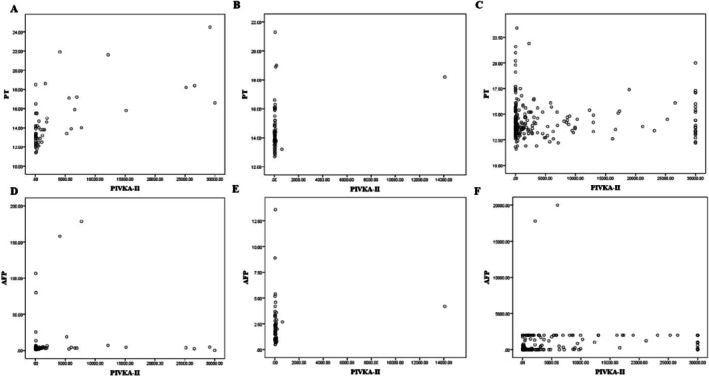
Correlation between PT, AFP, and PIVKA‐II in patients with OJ, Sepsis, and HCC. (A, D) OJ, (B, E) sepsis, (C, F) HCC. HCC, hepatocellular carcinoma; OJ, obstructive jaundice.

To evaluate whether markers of the severity of obstructive jaundice such as bile acids, total bilirubin, conjugated bilirubin, and coagulation function indicators (PT, INR, and APTT) were associated with serum PIVKA‐II, Pearson correlations were conducted between PIVKA‐II, AFP, and other measured parameters. Serum PIVKA‐II in patients with obstructive jaundice was significantly correlated with PT, INR, and APTT (*p* < 0.001, *p* < 0.001, and *p* = 0.002, respectively), as well as the severity of obstructive jaundice, as measured by serum total bilirubin and conjugated bilirubin (*p* = 0.018 and *p* = 0.049) (Table S1, Figure 2). In patients with sepsis, serum PIVKA‐II was positively correlated with PT, INR, and PCT (*p* = 0.029, *p* = 0.041, and *p <* 0.001, respectively). In contrast, no significant correlations were observed between serum AFP and coagulation function indicators. However, positive correlations were observed between AFP and ALT, AST, and bile acids (*p <* 0.001, *p =* 0.006, and *p* = 0.022, respectively) in obstructive jaundice, and between AFP and WBC, neutrophils, and CRP (*p <* 0.001, *p =* 0.001, and *p* = 0.005, respectively) in sepsis (Table S2, Figure 2). Notably, no significant correlations were found between serum PIVKA‐II and AFP in patients with obstructive jaundice and sepsis (*p* = 0.203 and *p* = 0.422). Serum PIVKA‐II was positively correlated with AFP, ALT, AST, ALP, and GGT in patients with HCC (*r* = 0.187, *p* = 0.004; *r* = 0.201, *p* = 0.002; *r* = 0.237, *p <* 0.001; *r* = 0.293, *p <* 0.001; and *r* = 0.364, *p <* 0.001, respectively), but not with PT, INR, and APTT (*r* = 0.029, *p =* 0.651; *r* = 0.019, *p =* 0.771; and *r* = 0.090, *p* = 0.162, respectively) (Table S3, Figure 2).

**FIGURE 3:**
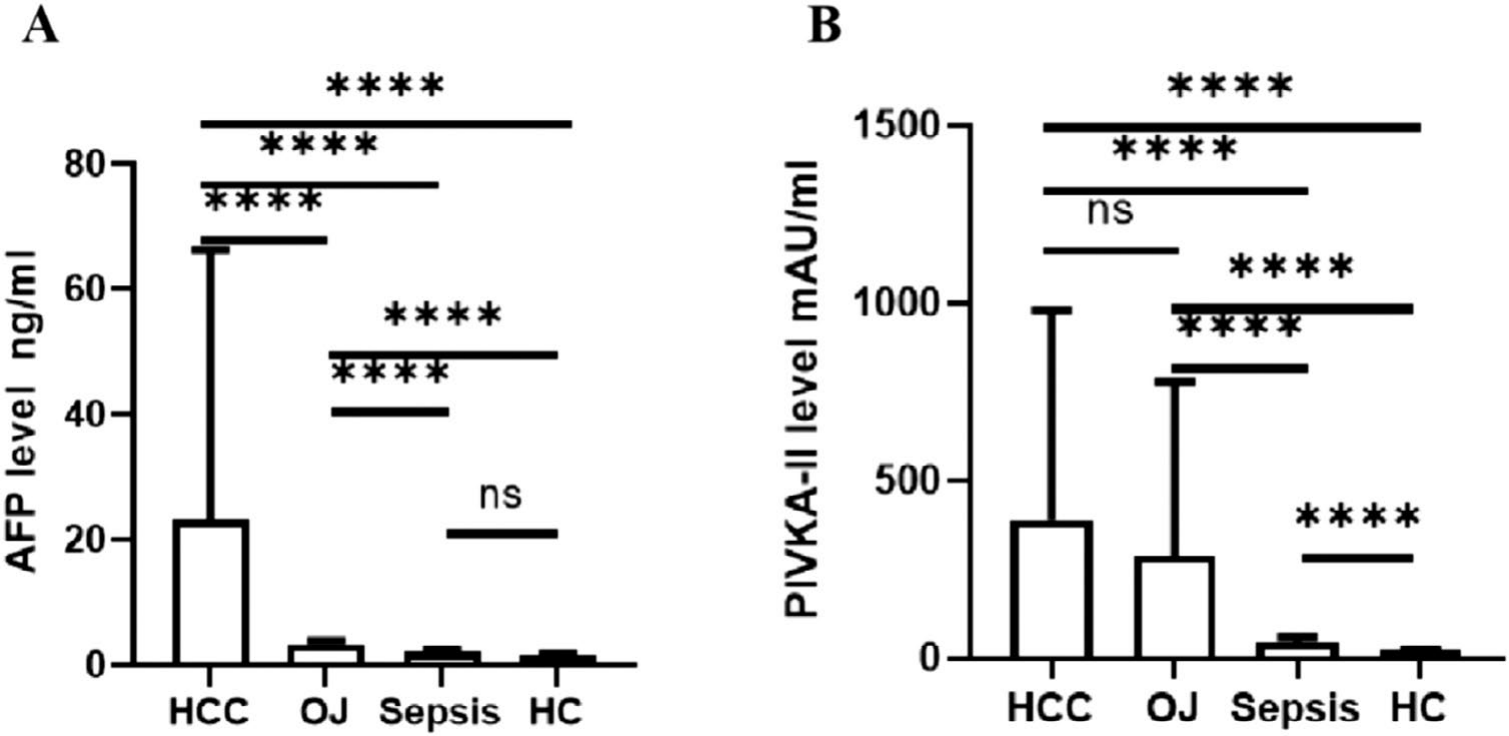
Comparison of serum tumor markers among HC, OJ, Sepsis, and HCC: (A) AFP, (B) PIVKA‐II. HC, healthy controls; HCC, hepatocellular carcinoma; OJ, obstructive jaundice.

As shown in Figure 3, there was no significant difference in PIVKA‐II concentration between the HCC and obstructive jaundice groups. However, statistical differences were observed in comparisons among all other groups (*p <* 0.001). Similarly, the AFP levels showed significant differences among all groups except for sepsis patients and healthy controls (*p <* 0.001). The seropositivity rate of serum PIVKA‐II was significantly higher than that of AFP across all three groups. Specifically, the seropositivity of PIVKA‐II in patients with obstructive jaundice was 82.76%, which was even higher than that observed in patients with primary hepatocellular carcinoma (75.10%) (Table 2).

**TABLE 2:** Comparison of positive rate of PIVKA‐II and AFP in the four groups.

Group		AFP	PIVKA‐II	*p*
Obstructive jaundice	Positive	8 (13.79%)	48 (82.76%)	< 0.001
	Negative	50 (86.21%)	10 (17.24%)	
Sepsis	Positive	2 (3.64%)	23 (41.82%)	< 0.001^a^
	Negative	53 (96.36%)	32 (58.18%)	
Primary hepatocellular	Positive	140 (58.09%)	181 (75.10%)	< 0.001
	Negative	101 (41.91%)	60 (24.90%)	
Healthy individuals	Positive	0 (0.00%)	2 (1.60%)	0.498^a^
	Negative	125 (100.00%)	123 (98.40%)	

*Note:* Data are presented as number (percentage).

Abbreviations: AFP, alpha‐fetoprotein; PIVKA‐II, protein induced by vitamin K absence or antagonist‐II.

^a^ Fisher's exact test (*E* < 5).

**FIGURE 4:**
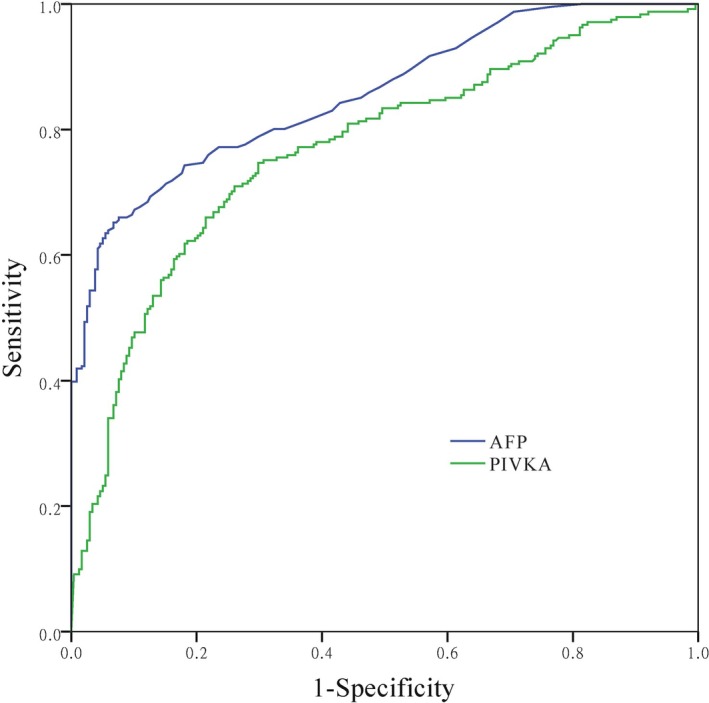
ROC curve of PIVKA‐II and AFP in the diagnosis of primary liver cancer under the interference of obstructive jaundice and sepsis.

The receiver operating characteristic (ROC) curve was used to determine the specificity and sensitivity of PIVKA‐II and AFP in diagnosing HCC under the interference of obstructive jaundice and sepsis (Figure 4). When the cut‐off for PIVKA‐II and AFP was 5.90 ng/mL and 42.17 mAU/mL, respectively, the Youden index (YI) for HCC was the best (Table 3). However, in the presence of sepsis and obstructive jaundice, the specificity, YI, positive likelihood ratio (+LR), and positive predictive value (PPV) of PIVKA‐II were inferior to AFP.

**TABLE 3:** Diagnostic performance of AFP and PIVKA‐II in HCC.

	Cut‐off	Sensitivity	Specificity	YI	PPV	NPV	+LR	−LR
AFP	5.90 ng/mL	63.49%	94.54%	58.03%	92.17%	71.88%	11.55	0.39
PIVKA‐II	42.17 mAU/mL	74.69%	70.17%	44.86%	71.71%	73.25%	2.51	0.36

Abbreviations: +LR, positive likelihood ratio; −LR, negative likelihood ratio; NPV, negative predictive value; PPV, positive predictive value; YI, Youden index.

In the obstructive jaundice cohort, there are 50 patients diagnosed with cancer and 40 cases are cholangiocarcinoma. There is no difference in PIVKA‐II levels and positive rates between benign obstructive jaundice and malignancy‐associated jaundice (*p* = 0.107 and *p* = 0.131) (Table 4). In patients with obstructive jaundice and sepsis, no significant difference in multivitamin uptake rates was found between the high‐PIVKA‐II and low‐PIVKA‐II groups (*p* = 0.922 and *p* = 0.162) (Table 5).

**TABLE 4:** Comparison of laboratory tests in patients between benign obstructive jaundice and malignancy‐associated jaundice.

Group	Benign obstructive jaundice (*n* = 8)	Malignancy‐associated jaundice (*n* = 50)	*F*/*Z*	*p*
Age	55.8 ± 12.2	62.3 ± 9.0	2.242	0.076
PIVKA‐II	45.81 (22.6–4505.4)	331.9 (65.8–1885.1)	−1.612	0.107
Positive rate	5/8 (62.5%)	43/50 (86.0%)	None	0.131
AFP	4.3 (2.4–11.2)	3.2 (2.2–4.7)	−0.835	0.404
PT	13.3 (12.5–15.3)	13.8 (12.8–15.5)	−0.474	0.636
INR	1.04 (1.00–1.24)	1.07 (0.96–1.30)	−0.282	0.778
APTT	35.6 (34.0–36.4)	37.4 (34.5–41.9)	−1.218	0.223
ALT	87 (39–205)	90 (49–149)	−0.023	0.982
AST	100 (61–117)	94 (56–139)	−0.259	0.795
GGT	500 (161–553)	305 (144–566)	−0.665	0.506
ALP	368 (280–525)	306 (171–475)	−1.082	0.279
Albumin	33.1 (31.1–36.3)	31.0 (28.0–35.3)	−1.240	0.215
Total bilirubin	111.5 (75.7–285.3)	219.4 (70.3–327.3)	−0.620	0.535
Conjugated bilirubin	74.2 (50.3–226.8)	125.9 (44.5–199.2)	−0.271	0.787
Acid biles	145.6 (87.9–187.4)	63.1 (13.7–156.1)	−1.939	0.052

*Note:* Data are presented as median and interquartile range (IQR).

Abbreviations: AFP, alpha‐fetoprotein; ALP, alkaline phosphatase; ALT, alanine aminotransferase; APTT, activated partial thromboplastin time; AST, aspartate aminotransferase; GGT, gamma‐glutamyl transferase; INR, international normalized ratio; PIVKA‐II, protein induced by vitamin K absence or antagonist‐II; PT, prothrombin time.

**TABLE 5:** Comparison of multivitamin supplementation in patients with obstructive jaundice and sepsis.

PIVKA‐II (ng/mL)		Multivitamin supplement		*p*
		Yes	No	
Obstructive jaundice	< 40	6/10 (60.00%)	4/10 (40.00%)	0.922^a^
	≥ 40 ng/mL	28/48 (58.33%)	20/48 (41.67%)	
Sepsis	< 40	12/32 (37.50%)	20/32 (62.50%)	0.162
	≥ 40	13/23 (56.52%)	10/23 (43.48%)	

^a^ Fisher's exact test (*E* < 5).

## 4 Discussion

Normal liver produces prothrombin under vitamin K action, but abnormal prothrombin is produced in patients with vitamin K deficiency or HCC. Protein induced by vitamin K absence or antagonist‐II, also called des‐c‐carboxy prothrombin is an abnormal form of prothrombin, resulting from disruptions in vitamin K metabolism or impaired γ‐glutamyl carboxylase activity in HCC, leading to the absence of γ‐carboxylated amino acid residues. PIVKA‐II is an additional serum marker for AFP‐negative or early‐stage HCC [13, 14].

In the present study, we found that positive rates of PIVKA‐II were significantly higher than those of AFP in patients with obstructive jaundice, sepsis and hepatocellular carcinoma in China, among which patients with obstructive jaundice had the highest positive rate of PIVKA‐II, and there was no difference in PIVKA‐II levels between obstructive jaundice cases and patients with HCC. Furthermore, serum PIVKA‐II was positively associated with AFP in patients with primary HCC, but no such association was observed in patients with obstructive jaundice and sepsis. This discrepancy may be due to the fact that both AFP and PIVKA‐II were associated with hepatocellular carcinoma in primary HCC patients, thereby establishing a correlation. In contrast, in patients with obstructive jaundice and sepsis, PIVKA‐II was primarily influenced by vitamin deficiency, especially in obstructive jaundice, while AFP was not associated with the vitamin and there was no link between them.

A pilot study in Italy showed that PIVKA‐II was positive in 24/26 (92.31%) of the pancreatic cancer patients, and its median serum concentration was also significantly higher than that of benign pancreatic disease [15]. Using two PIVKA‐II antibodies, Kanazumi et al. [16] found elevated PIVKA‐II levels were also observed in 10/27 (37.0%) and in 15/27 (55.6%) of the patients with pancreatic cancer, 7 of 16 (43.8%) and 9 of 16 (56.3%) of those with biliary tract cancer. The high levels of PIVKA‐II in patients with pancreatic cancer and biliary tract cancer were consistent with our findings.

A systematic review of 38 studies with 11,124 cases, revealed that PIVKA‐II alone was moderately accurate in detecting HCC (sensitivity 0.66, 95% CI 0.65–0.68; specificity 0.88, 95% CI 0.87–0.90; positive likelihood ratio [+LR] 7.13, 95% CI 5.73–8.87) [17]. However, in this study, the sensitivity, specificity, and +LR of PIVKA‐II in diagnosing hepatocellular carcinoma were 74.69%, 70.17%, and 2.51, respectively. The specificity and +LR in this study were inferior to measures in other studies. The reason was the increase in PIVKA‐II caused by sepsis and obstructive jaundice.

In this study, prolonged PTs in patients with obstructive jaundice and sepsis were significantly associated with elevated PIVKA‐II levels, but no such correlation was found in patients with primary hepatocellular carcinoma. Increased undercarboxylated prothrombin or decreased vitamin K levels have previously been identified in adults with cholestatic liver disease [18]. Similarly, Strople et al. [19] reported that PIVKA‐II was directly associated with PT and conjugated bilirubin in cholestatic liver disease, which strongly suggested that abnormal PIVKA‐II was the result of vitamin K deficiency. Excluding primary hepatocellular carcinoma, determination of prothrombin time and measurement of PIVKA levels (especially PIVKA‐II, or degamma‐carboxylated prothrombin) can be used to assess the severity of vitamin K deficiency [19, 20]. Impaired coagulation in patients with obstructive jaundice includes damage to liver cells, bacterial ectopia, and a lack of bile in the gut leading to poor absorption of vitamin K. Beyond septic and inflammatory complications, which contribute to hypercoagulability, the underlying pathology involves frequent antibiotic use, which disrupts the gut microbiota and inhibits the production of vitamin K_2_ by gut bacteria [20, 21, 22, 23]. Poor nutrition due to inadequate intake of hospital food, changed the intestinal microflora as a result of antibiotic treatment are the most common causes of vitamin K deficiency in sepsis patients [16, 20, 21, 22, 23, 24].

We found no significant difference in PIVKA‐II levels and positive rate between benign obstructive jaundice and malignancy‐associated jaundice. Therefore, we believe that the elevation of PIVKA‐II is not due to carcinoma similar to HCC. Tartaglione et al. [15] hypothesized that the PIVKA‐II produced by pancreatic cancer was due to the common foregut derivation of the liver and pancreas, their embryologic proximity and their demonstrated capability of mutual trans‐differentiation. However, Kanazumi et al. divided patients with cholangiocarcinoma and pancreatic cancer without HCC into two groups. One group received 20 mg of vitamin K_2_ administered intravenously daily for 21 days after surgery, while the other group had no vitamin K_2_. PIVKA‐II levels of both groups decreased gradually to the normal range within about 2 weeks after the operation. In the group of patients without vitamin K_2_, PIVKA‐II levels increased 14 days after surgery [16], which confirmed our conclusion that the increase in PIVKA‐II levels in non‐hepatocellular carcinoma patients was not related to cancer.

Serum PIVKA‐II elevation remained common in these patients, even with vitamin K therapy. As demonstrated in this study, despite multivitamin supplementation in 24/58 patients with obstructive jaundice and 25/55 patients with sepsis, the association between PIVKA‐II and coagulation markers remained robust. Previous studies have reported that different vitamin K treatment regimens have varying effects on coagulation function [23]. This suggests that even with supplementation, intake is not sufficient to overcome the absorptive defect in these patients and current practices of oral vitamin K supplementation may be inadequate to maintain vitamin K nutriture in cholestatic liver disease.

To date, the underlying mechanisms responsible for the overexpression of PIVKA‐II in HCC have not been fully elucidated. Given the complexity of prothrombin production, the overexpression of PIVKA‐II in HCC tissues is likely driven by a combination of mechanisms. Current evidence indicates that elevated PIVKA‐II levels correlate with factors such as the hypoxic microenvironment, reduced activity of γ‐glutamyl carboxylase, impaired vitamin K metabolism, and overexpression of the prothrombin precursor [25, 26]. Furthermore, previous studies have demonstrated that PIVKA‐II promoted tumor growth, invasion and metastasis by enhancing cellular proliferation, extracellular matrix synthesis, and tumor angiogenesis [27].

In the presence of taking vitamin K antagonists or vitamin K deficiency, a special PIVKA‐II variant, known as NX‐DCP, can be abnormally elevated in non‐HCC subjects [28, 29]. However, NX‐DCP and HCC‐derived PIVKA‐II can be distinguished based on the number of glutamic acid (Glu) residues. PIVKA‐II derived from HCC patients typically carried 6–9 Glu residues, whereas NX‐DCP contained 1–5 Glu residues. Although routine tests using MU‐3 antibodies cannot distinguish differences in Glu residues, serum NX‐DCP levels can be detected by P‐11 or P‐16 antibodies. Nonetheless, the high variability between different examination batches and high cost limited the clinical application of P‐11 or P‐16 antibodies [30]. Nevertheless, several limitations should be taken into consideration. This was a single‐center retrospective study, which may be prone to selection bias. Additionally, among the 58 patients with obstructive jaundice, there were only eight cases of benign obstructive jaundice. The number of included cases was relatively small, which led to a certain margin of error.

In conclusion, the PIVKA‐II produced by HCC may differ from that produced by obstructive jaundice and sepsis. Despite multivitamin supplementation, elevated serum PIVKA‐II levels suggesting ongoing vitamin K deficiency are commonly observed in patients with obstructive jaundice and sepsis. Given the abnormally elevated serum PIVKA‐II levels in patients without hepatocellular carcinoma, these results should be interpreted with caution in patients with hepatocellular carcinoma combined with these diseases.

## Ethics Statement

The study was approved by the Ethics Committee at Wuhan Union Hospital (the approved number 0887).

## Consent

The authors have nothing to report.

## Conflicts of Interest

The authors declare no conflicts of interest.

## Supporting information


**Table S1:** jcla70128‐sup‐0001‐TableS1.docx.

**Table S2:** jcla70128‐sup‐0002‐TableS2.docx.

**Table S3:** jcla70128‐sup‐0003‐TableS3.docx.

## Data Availability

The datasets generated and/or analyzed during the current study are not publicly available due to a laboratory confidentiality agreement, but are available from the corresponding author on reasonable request.

## References

[1] A. Forner , M. Reig , and J. Bruix , “Hepatocellular Carcinoma,” Lancet 391, no. 10127 (2018): 1301–1314. 29307467 10.1016/S0140-6736(18)30010-2

[2] N. Poté , F. Cauchy , M. Albuquerque , et al., “Performance of PIVKA‐II for Early Hepatocellular Carcinoma Diagnosis and Prediction of Microvascular Invasion,” Journal of Hepatology 62, no. 4 (2015): 848–854. 25450201 10.1016/j.jhep.2014.11.005

[3] European Association for the Study of the Liver and European Organisation for Research and Treatment of Cancer , “EASL‐EORTC Clinical Practice Guidelines: Management of Hepatocellular Carcinoma,” Journal of Hepatology 56, no. 4 (2012): 908–943. 22424438 10.1016/j.jhep.2011.12.001

[4] A. Singal , M. L. Volk , A. Waljee , et al., “Meta‐Analysis: Surveillance With Ultrasound for Early‐Stage Hepatocellular Carcinoma in Patients With Cirrhosis,” Alimentary Pharmacology & Therapeutics 30, no. 1 (2009): 37–47. 19392863 10.1111/j.1365-2036.2009.04014.x PMC6871653

[5] D. Y. Kim , B. N. Toan , C. K. Tan , et al., “Utility of Combining PIVKA‐II and AFP in the Surveillance and Monitoring of Hepatocellular Carcinoma in the Asia‐Pacific Region,” Clinical and Molecular Hepatology 29, no. 2 (2023): 277–292. 36710606 10.3350/cmh.2022.0212 PMC10121296

[6] Y. Inagaki , H. L. Xu , K. Hasegawa , et al., “Des‐Gamma Carboxyprothrombin in Patients With Hepatocellular Carcinoma and Liver Cirrhosis,” Journal of Digestive Diseases 12, no. 6 (2011): 481–488. 22118699 10.1111/j.1751-2980.2011.00521.x

[7] M. Kudo , N. Izumi , N. Kokudo , et al., “Management of Hepatocellular Carcinoma in Japan: Consensus‐Based Clinical Practice Guidelines Proposed by the Japan Society of Hepatology (JSH) 2010 Updated Version,” Digestive Diseases 29, no. 3 (2011): 339–364. 21829027 10.1159/000327577

[8] Y. Yamashita , E. Tsuijita , K. Takeishi , et al., “Predictors for Microinvasion of Small Hepatocellular Carcinoma ≤ 2 cm,” Annals of Surgical Oncology 19, no. 6 (2012): 2027–2034. 22203184 10.1245/s10434-011-2195-0

[9] M. Rodríguez‐Perálvarez , T. V. Luong , L. Andreana , T. Meyer , A. P. Dhillon , and A. K. Burroughs , “A Systematic Review of Microvascular Invasion in Hepatocel Lular Carcinoma: Diagnostic and Prognostic Variability,” Annals of Surgical Oncology 20, no. 1 (2013): 325–339. 23149850 10.1245/s10434-012-2513-1

[10] M. Kudo , “Urgent Global Need for PIVKA‐II and AFP‐L3 Measurements for Surveillance and Management of Hepatocellular Carcinoma,” Liver Cancer 13, no. 2 (2024): 113–118. 38751558 10.1159/000537897 PMC11095620

[11] A. G. Singal , N. Tayob , A. Mehta , et al., “GALAD Demonstrates High Sensitivity for HCC Surveillance in a Cohort of Patients With Cirrhosis,” Hepatology 75, no. 3 (2022): 541–549. 34618932 10.1002/hep.32185 PMC8844059

[12] M. Tatsumura , M. Kato , K. Takahashi , and T. Funayama , “A Case of Significant Coagulopathy due to Vitamin K Deficiency Caused by the Administration of Cefazolin and Rifampicin and Hyponutrition After a Postoperative Infection of the Lumbar Spine,” Cureus 16, no. 7 (2024): e64076. 39114205 10.7759/cureus.64076 PMC11305087

[13] P. R. Galle , F. Foerster , M. Kudo , et al., “Biology and Significance of Alpha‐Fetoprotein in Hepatocellular Carcinoma,” Liver International 39, no. 12 (2019): 2214–2229. 31436873 10.1111/liv.14223

[14] X. Xing , L. Cai , J. Ouyang , et al., “Proteomics‐Driven Noninvasive Screening of Circulating Serum Protein Panels for the Early Diagnosis of Hepatocellular Carcinoma,” Nature Communications 14, no. 1 (2023): 8392. 10.1038/s41467-023-44255-2 PMC10728065 38110372

[15] S. Tartaglione , I. Pecorella , S. R. Zarrillo , et al., “Protein Induced by Vitamin K Absence II (PIVKA‐II) as a Potential Serological Biomarker in Pancreatic Cancer: A Pilot Study,” Biochemia Medica 29, no. 2 (2019): 020707. 31223261 10.11613/BM.2019.020707 PMC6559614

[16] N. Kanazumi , S. Takeda , S. Inoue , et al., “PIVKA‐II During Perioperative Period in Patients With Hepato‐Biliary‐Pancreatic Diseases,” Hepato‐Gastroenterology 47, no. 36 (2000): 1695–1699. 11149034

[17] J. De , Y. Shen , J. Qin , L. Feng , Y. Wang , and L. Yang , “A Systematic Review of Des‐γ‐Carboxy Prothrombin for the Diagnosis of Primary Hepatocellular Carcinoma,” Medicine (Baltimore) 95, no. 17 (2016): e3448. 27124038 10.1097/MD.0000000000003448 PMC4998701

[18] K. V. Kowdley , M. J. Emond , J. A. Sadowski , and M. M. Kaplan , “Plasma Vitamin K1 Level Is Decreased in Primary Biliary Cirrhosis,” American Journal of Gastroenterology 92, no. 11 (1997): 2059–2061. 9362192

[19] J. Strople , G. Lovell , and J. Heubi , “Prevalence of Subclinical Vitamin K Deficiency in Cholestatic Liver Disease,” Journal of Pediatric Gastroenterology and Nutrition 49, no. 1 (2009): 78–84. 19502999 10.1097/MPG.0b013e31819a61ff

[20] V. Papadopoulos , D. Filippou , E. Manolis , and K. Mimidis , “Haemostasis Impairment in Patients With Obstructive Jaundice,” Journal of Gastrointestinal and Liver Diseases 16, no. 2 (2007): 177–186. 17592568

[21] P. Hoffman , “Vitamin K Deficiency Symptom to Diagnosis: An Evidence‐Based Guide,” in Symptom to Diagnosis: An Evidence‐Based Guide, 4e, ed. S. C. Stern , A. S. Cifu , and D. Altkorn (McGraw‐Hill Education, 2020).

[22] M. A. Kuzu , I. T. Kale , C. Cöl , A. Tekeli , A. Tanik , and C. Köksoy , “Obstructive Jaundice Promotes Bacterial Translocation in Humans,” Hepato‐Gastroenterology 46, no. 28 (1999): 2159–2164. 10521960

[23] D. Takahashi , N. Egami , M. Ochiai , et al., “Vitamin K Prophylaxis in Neonates: Comparing Two Different Oral Regimens,” Journal of Perinatology 44, no. 10 (2024): 1491–1495. 38678081 10.1038/s41372-024-01981-9

[24] D. R. Mager , P. L. McGee , K. N. Furuya , and E. A. Roberts , “Prevalence of Vitamin K Deficiency in Children With Mild to Moderate Chronic Liver Disease,” Journal of Pediatric Gastroenterology and Nutrition 42, no. 1 (2006): 71–76. 16385257 10.1097/01.mpg.0000189327.47150.58

[25] S. X. Cui , X. F. Yu , and X. J. Qu , “Roles and Signaling Pathways of Des‐γ‐ Carboxyprothrombin in the Progression of Hepatocellular Carcinoma,” Cancer Investigation 34, no. 9 (2016): 459–464. 27673353 10.1080/07357907.2016.1227445

[26] L. Dong , X. Qiu , F. Gao , K. Wang , and X. Xu , “Protein Induced by Vitamin K Absence or Antagonist II: Experience to Date and Future Directions,” Biochimica et Biophysica Acta Reviews on Cancer 1878, no. 6 (2023): 189016. 37944832 10.1016/j.bbcan.2023.189016

[27] Y. Yang , G. Li , Z. Lu , Y. Liu , J. Kong , and J. Liu , “Progression of Prothrombin Induced by Vitamin K Absence‐II in Hepatocellular Carcinoma,” Frontiers in Oncology 11 (2021): 726213. 34900676 10.3389/fonc.2021.726213 PMC8660097

[28] A. Nakao , Y. Suzuki , K. Isshiki , et al., “Clinical Evaluation of Plasma Abnormal Prothrombin (Des‐Gamma‐Carboxy Prothrombin) in Hepatobiliary Malignancies and Other Diseases,” American Journal of Gastroenterology 86, no. 1 (1991): 62–66. 1702578

[29] N. Rapp , V. M. Brandenburg , N. Kaesler , et al., “Hepatic and Vascular Vitamin K Status in Patients With High Cardiovascular Risk,” Nutrients 13, no. 10 (2021): 3490. 34684491 10.3390/nu13103490 PMC8539359

[30] A. Sumi , J. Akiba , S. Ogasawara , et al., “Des‐γ‐Carboxyprothrombin (DCP) and NX‐DCP Expressions and Their Relationship With Clinicopathological Features in Hepatocellular Carcinoma,” PLoS One 10, no. 3 (2015): e0118452. 25739032 10.1371/journal.pone.0118452 PMC4349810

