# Supplementary material for: Protein Induced by Vitamin K Absence or Antagonist‐II: Significantly Elevated in Obstructive Jaundice and Sepsis Patients Without Hepatocellular Carcinoma
Source: J Clin Lab Anal. 2025 Nov 13;39(24):e70128. doi: 10.1002/jcla.70128 (PMC12713562; doi:10.1002/jcla.70128)
Supplement: Supplementary file 1 — Table S1: jcla70128‐sup‐0001‐TableS1.docx. [file JCLA-39-e70128-s002.docx]

Table S1. Correlations of serum PIVKA-II and AFP with laboratory tests in patients with obstructive jaundice (n=58)

| Variable | PIVKA-II | | | AFP | | |
| --- | --- | --- | --- | --- | --- | --- |
|  | Pearson r | *P* | | Pearson r | *P* | |
| AFP | 0.170 | | 0.203 | 1 | | None |
| PIVKA-II | 1 | | None | 0.170 | | 0.203 |
| PT | 0.644 | | <0.001 | 0.192 | | 0.149 |
| INR | 0.683 | | <0.001 | 0.181 | | 0.174 |
| APTT | 0.391 | | 0.002 | 0.117 | | 0.383 |
| ALT | 0.005 | | 0.971 | 0.493 | | <0.001 |
| AST | 0.169 | | 0.204 | 0.358 | | 0.006 |
| GGT, | 0.022 | | 0.872 | -0.121 | | 0.366 |
| ALP | 0.185 | | 0.163 | -0.169 | | 0.206 |
| Albumin | -0.112 | | 0.403 | -0.147 | | 0.271 |
| Total bilirubin | 0.309 | | 0.018 | 0.138 | | 0.302 |
| Conjugated bilirubin | 0.260 | | 0.049 | 0.089 | | 0.504 |
| Bile acids | 0.042 | | 0.755 | 0.300 | | 0.022 |

PT, Prothrombin time; INR, International normalized ratio; APTT, Activated partial thromboplastin time; AFP, Alpha-fetoprotein; PIVKA-II, Protein induced by vitamin k absence or antagonist-II; ALT, Alanine aminotransferase; AST, Aspartate aminotransferase; GGT, Gamma-glutamyl transferase; ALP, Alkaline Phosphatase.

Data are presented as median and interquartile range (IQR).
