# Supplementary material for: Protein Induced by Vitamin K Absence or Antagonist‐II: Significantly Elevated in Obstructive Jaundice and Sepsis Patients Without Hepatocellular Carcinoma
Source: J Clin Lab Anal. 2025 Nov 13;39(24):e70128. doi: 10.1002/jcla.70128 (PMC12713562; doi:10.1002/jcla.70128)
Supplement: Supplementary file 2 — Table S2: jcla70128‐sup‐0002‐TableS2.docx. [file JCLA-39-e70128-s001.docx]

Table S2. Correlations of serum PIVKA-II and AFP with laboratory tests in patients with sepsis (n=55)

| Variable | PIVKA-II | | AFP | |
| --- | --- | --- | --- | --- |
|  | Pearson r | *P* | Pearson r | *P* |
| AFP | 0.110 | 0.422 | 1 | None |
| PIVKA-II | 1 | None | 0.110 | 0.422 |
| PT | 0.294 | 0.029 | -0.059 | 0.668 |
| INR | 0.276 | 0.041 | -0.051 | 0.713 |
| APTT | -0.003 | 0.986 | -0.106 | 0.439 |
| ALT | -0.065 | 0.635 | 0.041 | 0.767 |
| AST | -0.084 | 0.540 | 0.096 | 0.486 |
| WBC | 0.038 | 0.784 | -0.468 | <0.001 |
| Neutrophil | 0.054 | 0.694 | -0.442 | 0.001 |
| PCT | 0.562 | <0.001 | -0.079 | 0.567 |
| CRP | -0.042 | 0.764 | -0.377 | 0.005 |

PT, Prothrombin time; INR, International normalized ratio; APTT, Activated partial thromboplastin time; AFP, Alpha-fetoprotein; PIVKA-II, Protein induced by vitamin k absence or antagonist-II; ALT, Alanine aminotransferase; AST, Aspartate aminotransferase; GGT, Gamma-glutamyl transferase; ALP, Alkaline Phosphatase; CRP, C-reactive protein; PCT, Procalcitonin; WBC, White blood cell.

Data are presented as median and interquartile range (IQR).
