# Supplementary material for: Protein Induced by Vitamin K Absence or Antagonist‐II: Significantly Elevated in Obstructive Jaundice and Sepsis Patients Without Hepatocellular Carcinoma
Source: J Clin Lab Anal. 2025 Nov 13;39(24):e70128. doi: 10.1002/jcla.70128 (PMC12713562; doi:10.1002/jcla.70128)
Supplement: Supplementary file 3 — Table S3: jcla70128‐sup‐0003‐TableS3.docx. [file JCLA-39-e70128-s003.docx]

Table S3. Correlations of serum PIVKA-II and AFP with laboratory tests in patients with primary hepatocellular (n=241)

| Variable | PIVKA-II | | AFP | |
| --- | --- | --- | --- | --- |
|  | Pearson r | *P* | Pearson r | *P* |
| AFP | 0.187 | 0.004 | 1 | None |
| PIVKA-II | 1 | None | 0.187 | 0.004 |
| PT | 0.029 | 0.651 | -0.025 | 0.697 |
| INR | 0.019 | 0.771 | -0.015 | 0.812 |
| APTT | 0.090 | 0.162 | -0.008 | 0.896 |
| Total bilirubin | 0.080 | 0.219 | 0.065 | 0.316 |
| Conjugated bilirubin | 0.077 | 0.231 | 0.068 | 0.295 |
| ALT | 0.201 | 0.002 | 0.014 | 0.827 |
| AST | 0.237 | <0.001 | 0.027 | 0.679 |
| ALP | 0.293 | <0.001 | 0.099 | 0.126 |
| GGT | 0.364 | <0.001 | 0.120 | 0.062 |
| Acid biles | -0.047 | 0.464 | -0.008 | 0.906 |
| Albumin | -0.055 | 0.395 | -0.133 | 0.039 |
| WBC | 0.006 | 0.929 | 0.009 | 0.885 |
| Neutrophil | 0.108 | 0.095 | 0.042 | 0.516 |

PT, Prothrombin time; INR, International normalized ratio; APTT, Activated partial thromboplastin time; AFP, Alpha-fetoprotein; PIVKA-II, Protein induced by vitamin k absence or antagonist-II; ALT, Alanine aminotransferase; AST, Aspartate aminotransferase; GGT, Gamma-glutamyl transferase; ALP, Alkaline Phosphatase; WBC, White blood cell.

Data are presented as median and interquartile range (IQR).
